# Multiple Food-Animal-Borne Route in Transmission of Antibiotic-Resistant *Salmonella* Newport to Humans

**DOI:** 10.3389/fmicb.2018.00023

**Published:** 2018-01-23

**Authors:** Hang Pan, Narayan Paudyal, Xiaoliang Li, Weihuan Fang, Min Yue

**Affiliations:** ^1^CATG Microbiology & Food Safety Laboratory, Institute of Preventive Veterinary Medicine, College of Animal Sciences of Zhejiang University, Hangzhou, China; ^2^Zhejiang Provincial Key Laboratory of Preventive Veterinary Medicine, Hangzhou, China

**Keywords:** *Salmonella* Newport, antibiotic resistant, food animal, transmission, population structure, random forest

## Abstract

Characterization of transmission routes of *Salmonella* among various food-animal reservoirs and their antibiogram is crucial for appropriate intervention and medical treatment. Here, we analyzed 3728 *Salmonella enterica* serovar Newport (*S.* Newport) isolates collected from various food-animals, retail meats and humans in the United States between 1996 and 2015, based on their minimum inhibitory concentration (MIC) toward 27 antibiotics. Random Forest and Hierarchical Clustering statistic was used to group the isolates according to their MICs. Classification and Regression Tree (CART) analysis was used to identify the appropriate antibiotic and its cut-off value between human- and animal-population. Two distinct populations were revealed based on the MICs of individual strain by both methods, with the animal population having significantly higher MICs which correlates to antibiotic-resistance (AR) phenotype. Only ∼9.7% (267/2763) human isolates could be attributed to food–animal origins. Furthermore, the isolates of animal origin had less diverse antibiogram than human isolates (*P* < 0.001), suggesting multiple sources involved in human infections. CART identified trimethoprim-sulfamethoxazole to be the best classifier for differentiating the animal and human isolates. Additionally, two typical AR patterns, MDR-Amp and Tet-SDR dominant in bovine- or turkey-population, were identified, indicating that distinct food-animal sources could be involved in human infections. The AR analysis suggested fluoroquinolones (i.e., ciprofloxacin), but not extended-spectrum cephalosporins (i.e., ceftriaxone, cefoxitin), is the adaptive choice for empirical therapy. Antibiotic-resistant *S.* Newport from humans has multiple origins, with distinct food-animal-borne route contributing to a significant proportion of heterogeneous isolates.

## Introduction

Foodborne infection is an important cause of morbidity and mortality worldwide (Kirk et al., 2015). Bacteria, including *Salmonella*, are the leading cause of foodborne illnesses. Although most human cases of foodborne infections are mild, appropriate antibiotics treatment are essential for life-threatening cases, particularly in very young or elderly patients and in patients with systemic infection. However, the antibiotic-resistant, especially multi-drug resistant, virulent clones can compromise antibiotic treatment, and increase the chances of severe complications due to post-antibiotic and/or infection-induced dysbiosis (Baumler and Sperandio, 2016; Faber et al., 2016; Yue, 2016). Thus, the emerging of *Salmonella*, particularly those virulent antibiotic-resistant clones, poses the most significant threat to public health and food safety.

*Salmonella* includes numerous serovars each having different capabilities to colonize and/or infect its corresponding hosts or niches. *Salmonella enterica* serovar Newport (*S.* Newport), one of third top-ranked serovar for human infections in the United States, are generally recognized as one of the major serovar for cattle (Afema et al., 2015; Mollenkopf et al., 2017). Importantly, *S.* Newport are capable of long-term survival in the manure environments (You et al., 2006), which can serve as the alternative evolving reservoirs for both animal and human infections. Most intriguingly, *S.* Newport can colonize, invade the plant tissue and possess high resistance during food processing, which support the evidence that long-term persistence in various environment (Cooley et al., 2003; Barak et al., 2005; Gorbatsevich et al., 2013; Zheng et al., 2013; Han and Micallef, 2014). Recent recurring incidences of *S.* Newport outbreaks due to fresh produce is a typical example suggest an emerging environmental route for human infections (Li et al., 2014; Bell et al., 2015; Luo et al., 2015). Additionally, wild animal and its associated environment can provide the ecological reservoirs for *S.* Newport persistence (Gruszynski et al., 2014a,b), with the evidence of frequently food samplings in fish (Seepersadsingh and Adesiyun, 2003), oyster (Brands et al., 2005a,b; Brillhart and Joens, 2011; Morrison et al., 2011; Lo et al., 2017). It is not clear how diversified niches or non-human hosts contribute to *S.* Newport human infections.

*Salmonella* Newport was previously indicated as a polyphyletic serovar, which was confirmed by whole genome sequencing data and MLST analysis (Sangal et al., 2010; Zheng et al., 2017). Three lineages were defined after analyzing 384 Newport isolates from various hosts and geographical niches by MLST, with lineage-I for human, lineage-II for non-human hosts (typically avian, bovine, equine, swine, and reptiles), lineage-III for a mixture of human and chicken. Additionally, antibiotic resistance profiles between lineage-I and -II were different, which indicated that human infections from lineage-I were less likely from animal reservoirs in lineage-II. However, few studies focus on transmission through the food chain and little is known the relationship among *S.* Newport isolates via food-chain samplings, various food-animals, and humans.

Generally, *S.* Newport is transmitted to the humans via consumption of contaminated foods of animal origin, such as beef, pork, poultry, and milk, or of non-animal origins such as vegetables and other fresh produce (Varma et al., 2006; Afema et al., 2015; Gomba et al., 2016). Few investigations focus on how different transmission routes (i.e., various food animal origins, i.e., bovine, swine, chicken, turkey) are involved in human infections, especially, in the transmission of antibiotic-resistant *Salmonella* to humans. Here, by analyzing the minimum inhibitory concentration (MIC) values of a range of 27 antibiotics for 3728 *S.* Newport strains, sampling from various food animals, their according retail meat and human across two decades in the United States, we try to evaluate the importance of different food-animal-borne routes, and other possible sources, for transmission of antibiotic resistance.

## Materials and Methods

### *Salmonella* Strain Collection

We queried the computerized United States National Antimicrobial Resistance Monitoring System (NARMS) for Enteric Bacteria database of submitted specimens from state health departments to identify *S.* Newport isolates from January 1, 1996 through January 31, 2017. There were a total of 3728 *S.* Newport strains isolated from humans (2763), animals (901), and retail-meats (64). The *S*. Newport strains of animal origins were mainly from bovines (726) and less frequently from turkeys (139), chickens (60), and pigs (40). The animal strains were categorized as live animals (bovine, swine, chicken, and turkey) and meat (ground-beef, GB; chicken-breasts, CB; pork-chop, PC; ground-turkey, GT). The procedures for *Salmonella* isolation, confirmation, and serotyping were conducted as previously described (Rankin et al., 2002, 2005; Yue et al., 2012).

### Minimum Inhibitory Concentration (MIC) Assay

The antimicrobial MICs were obtained by the agar dilution method and interpreted according to the guidelines recommended by the Clinical and Laboratory Standards Institute (CLSI). The MICs were tested for a range of 27 antibiotics, the names, abbreviations and the cut-off of which as used in the NARMS system and our analyses are listed in **Table 1**.

**Table 1:** Details of the antibiotics used in the MIC (isolates with MIC value lower than the cut-off were regarded as sensitive whereas those higher than the cut-off were regarded as resistant).

CLSI class	Name	NARMSCode	Cut off	Remarks
Aminoglycosides	Amikacin	AMI	≥64	
	Apramycin	APR		
	Gentamicin	GEN^∗^	≥16	
	Kanamycin	KAN^#^	≥64	
	Streptomycin	STR^∗^		
B-Lactamase inhibitors	Amoxicillin-clavulanic acid	AMC^∗^	≥32/6	
	Piperacillin-tazobactam	PTZ	≥128/4	
Cephems	Cephalothin	CEP	≥32	1st Gen cephalosporin
	Cefoxitin	FOX^#^	≥32	2nd Gen cephalosporin
	Ceftriaxone	AXO^∗#^	≥4	3rd Gen cephalosporin
	Ceftiofur	TIO^∗^	≥8	
	Ceftazidime	CAZ	≥16	
	Cefotaxime	CTX	≥4	
	Cefotaxime/clavulanic acid	CTC		
	Cefquinome	CEQ		4th Gen cephalosporin
	Cefepime	FEP	≥16	
Folate pathway inhibitors	Sulfamethoxazole	SMX	≥512	
	Sulfisoxazole	FIS	≥512	
	Sulfamethoxazole-trimethoprim	COT^∗#^	≥4/76	
Macrolides	Azithromycin	AZM^#^	≥32	
Monobactam	Aztreonam	ATM	≥16	
Penems	Imipenem	IMI^#^	≥4	
Penicillin	Ampicillin	AMP^∗^	≥32	
Phenicol	Chloramphenicol	CHL^∗#^	≥32	
Quinolone	Ciprofloxacin	CIP^∗#^	≥4	
	Nalidixic acid	NAL^∗#^	≥32	
Tetracycline	Tetracycline	TET^∗^	≥16	

*^∗^Represents the core of 11 antibiotics for which the data were consistently available throughout the period of sample collection and are analyzed likewise in **Figure 3**; ^#^Represents the subset of nine antibiotics most commonly used in human medicine and are analyzed likewise in **Figure 4**.*

### Statistical Analysis

Random Forest (classification of 1000 support tree with out of bag data used for testing) and Hierarchical Clustering (1000 bootstrapping with Manhattan distance to generate an average linkage clustering tree) statistics were used to differentiate *Salmonella* population according to their MIC profiles. To prioritize probable causal association, a Random Forest classification was performed using Random Forest package 4.6 from the Bioconductor. The resulting classification model consists of 1000 decision trees trained on candidate variants (antibiotics) that were determined to be predictive of resistance. Classification and Regression Tree, named CART (with a limited splitting node 500) statistic was used to identify the best classifier (antibiotic and its cut-off) for animals’ or humans’ population. Random Forest and CART, both as predictive analytics, were used to investigate either multidimensional scaling pattern among different population by machine-learning technique, or determine the most “important” variables based on explanatory classification power by data-mining technique. Additional comparative analysis of antibiotic-resistant profiles for strains of various host/source populations was conducted by multinomial logistic regression as described previously (Afema et al., 2015).

## Results

### Human- and Animal-Associated Clusters

**FIGURE 1:**
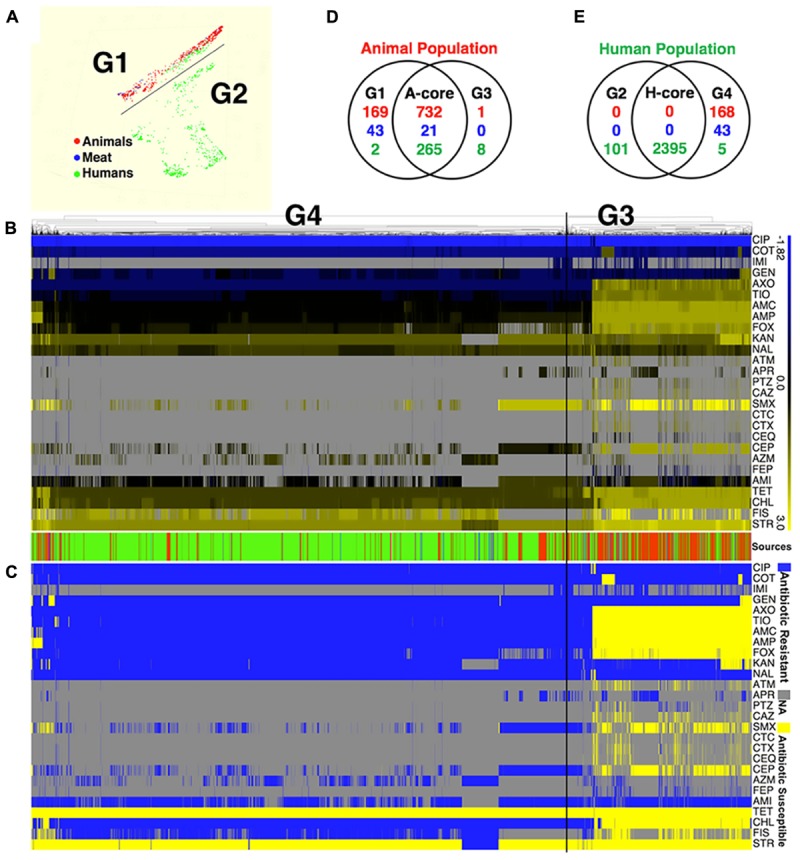
The population diversity of 3728 *S.* Newport isolates, sampling from human, bovine, porcine, chicken, and turkey. **(A)** Population diversity of *S.* Newport grouped in multidimensional scaling plot by RandomForest. The MIC value of 27 examined antibiotics for each strains were used to group different population. Two distinct population, G1 and G2, were detected. Each dot represents individual isolate, with colors indicating host origins. **(B)** Population diversity of *S.* Newport grouped by hierarchical clustering. A hierarchical tree with 200 bootstrapping, by using the MIC value of 27 antibiotics, was used to group different population. Two distinct population, G3 (left) and G4 (right), were detected. The color of heatmap, from blue (–1.82) to yellow (3), shows the log value of MIC of individual isolate for each antibiotic. The color of sources (last row) shows the origin of individual strain, with red (animal), blue (meat), and green (human); gray color indicates strains without MIC value. The source bar is shared by **(B,C)**. **(C)** The antibiogram for individual strains are shown, with yellow indicating the susceptibility, and blue indicating the resistance, based on the MICs interpreted by the CLSI-2015 standards. Gray color indicates strains without MIC value. **(D)** The Venn diagram for correlation of animal-core group (red) by two animal population G1 and G3. Animal core group is defined as the cluster of those isolates that have been derived from the samples originating in the live animals or animal meat. **(E)** The Venn diagram for correlation of human-core group (green) by two human population G2 and G4. Human core group is defined as the cluster of those isolates originating from the human samples. For the details on the antibiotics used in the MIC as well the corresponding abbreviations shown in this figure, please refer to **Table 1**.

**FIGURE 2:**
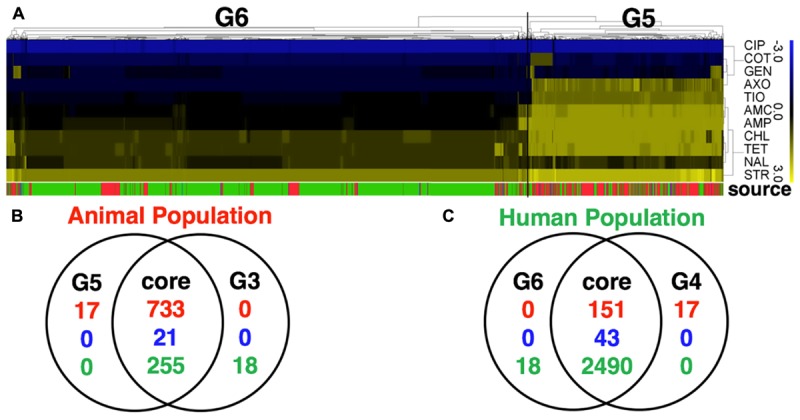
Comparative analysis of *S.* Newport population with a full panel of 27 antibiotics tested for all strains (with partially data missing) and a subset of 11 selected antibiotics tested for all strains (with no missing data). **(A)** The heatmap of MIC value of a subset of 11 core antibiotics for 3728 *S.* Newport isolates. A hierarchical tree with 200 bootstrapping, by using the MIC value of eleven core antibiotics, was used to group different population. Two populations detected, named G5 (isolates of animal origin) and G6 (isolates of human origin). **(B,C)** The Venn diagram showing the comparative difference between two groups, G5 (11 antibiotics) and G3 (27 antibiotics) of animal origin or between two groups, G6 (11 antibiotics) and G4 (27 antibiotics) of human origin. The figures show a very high consistency for the animal- and human-associated group, indicating that these two datasets for grouping *S.* Newport population have minor differences. The color of source shows the origin of individual strain, with red (animal), blue (meat), and green (human).

Application of two different approaches to analyze the available data revealed that *S.* Newport strains of animal and human were found to originate in distinct populations (clusters). Random Forest used MIC values to graph and project the population diversity of *S.* Newport into two populations/groups. One group (G1, **Figure 1A**) included all strains of food animal origins (livestock and retail-meat isolates), as well as ∼9.7% (267/2763) human isolates (green in G1 **Figure 1A**). The other group (G2, **Figure 1A**), included solely of human strains with a very diverse antibiotic-resistant profiles. A hierarchical tree, constructed using the log values of the MICs for 27 antibiotics, revealed the diversity within *S.* Newport population, which were further grouped into G3 and G4 (**Figure 1B**). In **Figure 1C**, G3 and G4 were found to have distinct MIC value and antibiotic-resistant phenotype, with antibiotic resistance (in blue in **Figure 1C**) and antibiotic susceptibility (yellow in **Figure 1C**), when interpreted by the cut-off of CLSI. In **Figures 1B,C**, group G3 (animal core group, red color designed by source in between **Figures 1B,C**) included a larger number of animal isolates (*n* = 733) as compared to only 168 isolates in G4 (human core group, green color designed by source in between **Figures 1B,C**). In contrast, the majority of human isolates (2400/2763) were seen as set G4, which also included 273 human isolates in G3 (**Figure 1B**, indicated in green in source). The consistency between the two statistical approaches was corroborated by, the Venn diagram (**Figures 1D,E**), in which a number of strains were shared either by animal- or human-core group. ∼91.8% human strains belonging to the human-core had a very low MICs with rare antibiotic-resistant phenotype (**Figure 1C**), while 1018 animal-core strains had multiple origins, including the majority of animal (732), and a small fraction of human (265) and retail-meat (21). The animal and human groups were also evaluated further by using a subset of 11 antibiotics that were consistently available for these isolates examined for all strains (**Figure 2**).

### Antibiotic MICs Differentiate Animal-Associated Subgroups

**FIGURE 3:**
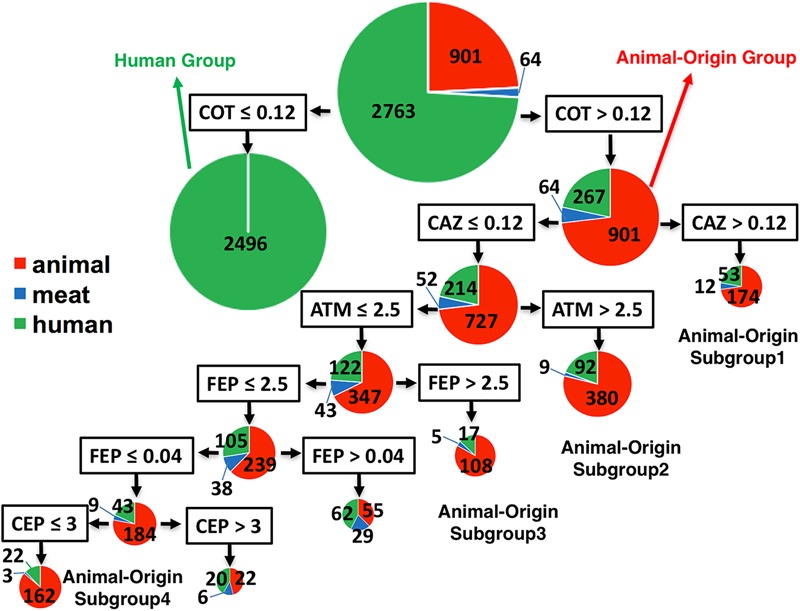
The classifier for differentiation of *S.* Newport isolates from animal, retail-meat or human. The 3728 isolates were mostly from human (green), and animal (red), including bovine, porcine, chicken and turkey, and retail-meat (blue) accordingly to the four animals. The best classifier for the differentiation of animal or human is trimethoprim-sulfamethoxazole (COT) with the cut-off 0.12. The isolates with COT value no more than 0.12 are all human isolates, while the later part with over 78% isolates having an animal-origin, indicated as animal-origin group (901 of animal, 64 of retail-meat, and 267 of human). Accordingly, ceftazidime (CAZ) with a cut-off 0.12, aztreonam (ATM) with a cut-off 2.5, cefepime (FEP) with a cut-off 2.5, cefepime (FEP) with a cut-off 0.04, and cephalothin (CEP) with a cut-off 3, can further divide the animal-origin group into several sub-clusters.

Using MICs as quantitative values to classify the two populations by CART analysis, some antibiotic combinations were detected to be associated with food animal population. Trimethoprim-sulfamethoxazole (COT, also known as TMP-SMZ) was predicted as the best classifier for the animal- or human-populations. ∼90% (2496/2763) human isolates had a COT cut-off ≤ 0.12 (**Figure 3**), suggesting that human isolates belonging to G2 were very susceptible (CLSI cut-off ≥ 4). Interestingly, for all isolates with cut-off > 0.12 (containing partially susceptible and all resistant), there were several statistically significant, distinct subgroups within food animal origins. These subgroups also carried supplementary antibiotic-resistant phenotype which made them unique. Subgroup-1 showed resistance to third-generation cephalosporin, ceftazidime (cut-off: ≥ 16) as an additional classifier (MIC of CAZ was > 24). Subgroup-2 had an additional classifier of aztreonam with MIC > 2.5, which is below the cut-off value of ≥ 16. Subgroup-3 also had additional unique classifier of cefepime, a fourth-generation cephalosporin with MIC > 0.09, below the cut-off of ≥ 16. Subgroup-4 had a sensitive phenotype to cephalothin (a first-generation cephalosporin). All those animal-associated subgroups shared a common feature – generally resistant to beta-lactam cephalosporins and represented themselves as a distinct group for human infections **Figure 1C**.

### Bovine- and Turkey-Associated Antibiotic-Resistance Patterns

**FIGURE 4:**
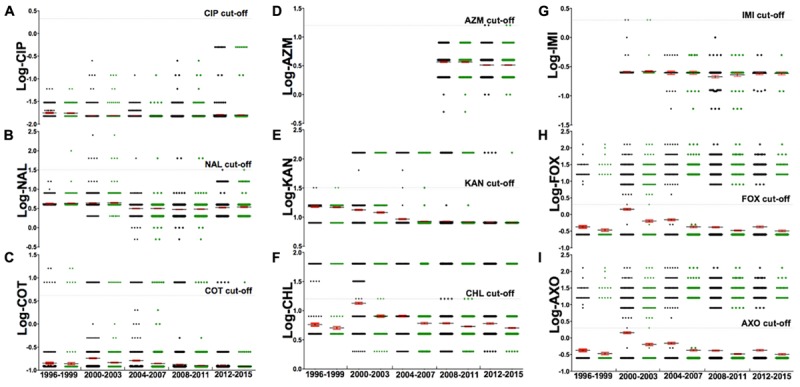
The analysis of a subset of nine antibiotics [**(A)** Ciprofloxacin, **(B)** Nalidixic acid, **(C)** Sulfa-trimethoprim, **(D)** Azithromycin, **(E)** Kanamycin, **(F)** Chloramphenicol, **(G)** Imipenem, **(H)** Cefoxitin, **(I)** Ceftriaxone] for their resistance, from 1996 to 2015. The gray dash indicates the CLSI cut-off for the corresponding antibiotic, with each dot representing log-MIC value of individual strains. Markers below the gray dash indicate sensitive MIC while those above the dash indicate resistant MIC. This subset of nine antibiotics were chosen from the set of 27 antibiotics because these nine represent a panel of drugs that are most commonly used in human medicine against non-typhoidal salmonella infections. An adjusted suggestion for antibiotics of empirical therapy is ciprofloxacin, azithromycin, and imipenem. Details of these antibiotics are described in **Table 1**.

The data of MIC assay, when profiled as resistance or susceptibility, based on the cut off values as presented in **Table 1**, resulted in generation of 149 antibiotic-resistant profiles (Supplementary Table 1), with humans’ having the most diverse antibiotic-resistant profiles (*P* < 0.001). Additionally, human isolates had the most (*n* = 62) of unique antibiotic-resistant profiles, compared to bovine (*n* = 36) and non-bovine (*n* = 14) isolates (**Figure 4**). Moreover, ∼94% (3503/3728) of strains belongs to 11 core antibiotic-resistant profiles, which could further be grouped into one commonly recognized profile MDR-Amp, and newly defined Tet-SDR (single drug resistance with resistant to tetracycline). When segregating the strains based on their core antibiotic-resistant profiles (MDR-Amp vs. Tet-SDR), we detected a statistically significant difference between bovine and human (unpaired *t*-test, *P* < 0.001). Poultry, particularly turkey, and human strains had highly correlated antibiotic-resistant profiles (Pearson correlation, *P* < 0.001). Significant differences in the dominant antibiotic-resistant profiles, between bovine and turkey, serve as an indicator of distinct reservoir for human infections.

## Discussion

Antibiotic resistance is one of most significant threats around the world, and it is clear that hospital- and community-acquired diseases play a key role in dissemination of antibiotic resistance (Laxminarayan et al., 2013; Bryce et al., 2016; Holmes et al., 2016). There are increasingly compelling evidences to show that use of antimicrobials in agricultural activities is another driver in selection of antibiotic-resistant bacteria (Miller et al., 2013; Angelo et al., 2015). Therefore, the exact contribution and attribution of antibiotic resistance in pathogenic bacteria of significance to human is an open issue with divided and sometimes contrasting opinions (Phillips et al., 2004; Founou et al., 2016; Lekshmi et al., 2017).

In spite of having plenty of information and hence the data on point estimates of foodborne *Salmonella*, relatively fewer studies that focus on the transmission of antibiotic resistant pathogens the food-chain are available. Still, relatively less is known about how *Salmonella* from various food animals and food-chain contributes to human infections, particularly for the dissemination of antibiotic-resistance. *S*. Newport, a massive colonizer in various livestock, wild animals, plants, and with long-term survival abilities, can serve an excellent model to investigate above questions.

### Limited Role of Food Animals in *S.* Newport Transmission to Humans

When using high-resolution antibiotic MIC profile, approximately 9.6% of the isolates from human and food animals showed similar antibiotic-resistant profile. This result, also could be verified by the fact that multi-drug resistance in human Salmonellosis was consistent at ∼10% for the past decade (CDC, 2016). Even though as early as 1973 food commodities like alfa-alfa sprouts were initially implicated as a vehicle for foodborne illness, it was not until the late 1990s when the epidemiologists actually started to investigate on fresh-produce-borne infections (Van Beneden et al., 1999). A recent investigation based on outbreak data suggested that fresh produce was attributed to 46% of human illnesses in the United States (Painter et al., 2013). Sporadic and outbreak-associated foodborne illness was suggested to be very similar, particularly in *Salmonella*, further confirming fresh produce is becoming an important risk factor for human Salmonellosis (Gomba et al., 2016). Furthermore, most of fresh produce isolates were susceptible to beta-lactam cephalosporins, fluoroquinolones, aminoglycosides, i.e., gentamicin and tetracyclines. Indeed, the diversity of *S.* Newport in G2 and G4 (in **Figures 1C,D**) with low MIC values (hence increased susceptibility) further support that *S.* Newport isolates for human infections are from a variety of origins, other than food animals. The divided population in animals 9.6% (with isolates having similar MIC profiles to humans) vs. 90.6% (isolates with MIC profiles different to those of humans), in current dataset, is suggestive that most of human infections caused by *S.* Newport are non-food-animal origins. Additional independent studies, investigating similar parameters on serovar Typhimurium and Enteritidis, supported the same trend (*unpublished data*).

Genetic and functional studies have also suggested that animal-origin *S.* Newport are distinct to those from other sources, including humans’. In 1988, different clonal lineages that vary in terms of their virulence, host range, and some other aspects of pathogen biology was defined by enzyme electrophoresis analysis. There are two major clonal lineages; one of them occurring predominantly in the humans and the other associated primarily with the domesticated animals but for other serotypes such distinction as not evident (Beltran et al., 1988). After more than 25 years, MLST analysis has revealed that Lineage-I represents typical human isolates, with much larger diversity and less antibiotic resistance phenotype, while lineage-II represents typical non-human, animal and some amphibians originated *S.* Newport. Similarly, food animal isolates are highly resistant when compared to the rest of the groups (Sangal et al., 2010). Similar to these reports our analyses also show a strong differential host association of *S.* Newport with either humans or animals, with only a small portion being common between these two larger chunks. Additional functional experiments in our group, and genomic analysis suggested that *S.* Newport of different lineages carried distinct genetic signatures and behaved with dramatically different pathogenic features (Yue et al., 2012, 2015; Yue and Schifferli, 2014; De Masi et al., 2017; Zheng et al., 2017), further supporting the distinct populations in *S.* Newport. Additional large-scale population analysis of *S.* Newport with a focus on other sources such as many sea animals and various of wild-caught or farm-reared amphibians (Deekshit et al., 2012, 2015; Goupil et al., 2012; Sylvester et al., 2014; Nowakiewicz et al., 2015; Ives et al., 2016; Nguyen et al., 2016; Ribas and Poonlaphdecha, 2017) needs further investigations.

### *S.* Newport from Bovines and Poultry Reservoir with Distinct Antibiotic Resistance Phenotype

When using low-resolution antibiotic resistance or susceptibility results, the bovine- and turkey-associated antibiotic-resistant profiles were detected. It is generally accepted that bovines are the primary reservoir for *S.* Newport, and accordingly to some human infections. To distinguish this type of resistance from other multidrug-resistant strains, these strains are referred to as Newport MDR-AmpC. The AmpC-type enzymes produced by *bla_CMY_* gene, confer resistance to penicillin-inhibitor combinations (e.g., amoxicillin/clavulanate), cephamycins (e.g., cefoxitin), as well as to the expanded-spectrum cephalosporins (e.g., ceftiofur and ceftriaxone). That is why, treatment of these infections with ceftriaxone could be ineffective (Centers for Disease Control and Prevention, 2002). During 1995–2010, an increasing trend for *S.* Newport human infections were reported, and most of human infections had a tangible connection to bovine or beef consumptions (Gupta et al., 2003; Greene et al., 2008). The key feature of bovine-origin isolates, including several bovine or equine outbreak studies suggest a common antibiotic resistance feature of beta-lactam and cephalosporins resistance, with relative genetic relationship (Cummings et al., 2010).

Few studies suggest that poultry are involved with *S.* Newport infections. Unexpectedly, our analysis showed that the Tet-SDR profile is found mainly in poultry, especially the turkeys. A few studies in retail turkey meat and ready-to-eat turkey suggest to have detected less resistant *Salmonella* (Fakhr et al., 2006; Khaitsa et al., 2007). Another study has also suggested that the age of the birds destined for slaughter affects the resistance pattern to the various antibiotics with younger birds as compared to the older ones being more resistant to the drugs such as tetracyclines and sulfisoxazole (Santos et al., 2007). Majority of turkey-origin isolates carried Tet-SDR, indicating previously underappreciated antibiotic-resistant reservoir for human infections. This result coincided with increasing poultry consumption and a large proportion of SDR isolates found in humans (Varma et al., 2006).

### Antibiotic Choice and Important Classifiers of a Sub-population

An updated knowledge of optimal antibiotic choice for severe infections is the priority in human medicine. Animal-borne infection could significantly impact the clinical outcome since the majority of multi-drug resistant strains are revealed to have an animal-origin. And because very rare (< 1%) human isolates of *S.* Newport are resistant, COT could be an ideal antibiotic to treat human *Salmonella* infections, with special caution for the case with the known history of animal-origin. Accordingly, COT should be prescribed cautiously for treating animal *S.* Newport infections (∼7.5% resistance). The current antibiotic regimen recommended for prescription by a physician against human Salmonellosis include quinolones, macrolides, and third-generation cephalosporins (Guerrant et al., 2001). However, as most of the animal-origin isolates have high-level resistance to cephalosporin (i.e., ceftriaxone, cefoxitin), the recommended cephalosporins usage, could be a big concern when treating *Salmonella* of animal-origin, either infecting humans or animals. Thus, empirical usage of ceftriaxone and cefoxitin should be modified, but imipenem could be an alternative candidate (**Figure 4**). Ciprofloxacin still can serve for treatment. Both tetracycline (∼99%) and streptomycin (∼95%) showed high-level resistance (**Figure 1C**), suggesting these antibiotics should be avoided for usage in clinic therapy or as animal-growth promoters.

Trimethoprim-sulfamethoxazole, is a combination of two antimicrobial agents that act synergistically by blocking the making of folate by bacteria. In our study, COT is the best classifier for differentiation of food animal- or human-population, which is also the case in other top-ranked serovars, Typhimurium, and Enteritidis (*unpublished data*). In United States agricultural activities, COT was widely used for therapeutic purpose administered through water, in contrast to growth-promoting use, where antibiotics are added in feed. However, in human populations, due to its higher incidents of adverse effects, including allergic response, COT usage has been restricted in many countries, including United States, to very specific circumstance (Economou and Gousia, 2015). These usage difference reflects partially the difference in acceleration of antibiotic resistance of two populations. For companion animal, COT was widely used in treatment of many bacterial infections of lungs, urinary tract, skin and gastrointestinal organs. It is likely that companion animal is another important reservoir for transmission of COT resistant *Salmonella* to human (Van Immerseel et al., 2004), which warranted further investigations.

Another shared feature in food animals is that *S.* Newport isolates are more likely resistant to various cephalosporins. Current studies also confirmed that *S.* Newport samplings from cattle and ground beef exhibited 64.3% (Munoz-Vargas et al., 2017) and 40% (Iwamoto et al., 2017) resistance to extend-spectrum cephalosporins, respectively. The CEP, FEP, and CAZ, belonging to cephem class, and aztreonam (ATM) belonging to monobactam class, are the detected classifiers in animal sub-populations. The preferential resistance in different sub-populations likely reflects various beta-lactamase genes, i.e., *bla_CMY_, bla_OXA_, bla_SHV_*, carried by *S.* Newport isolates, which were partial associated with plasmids types in different strains (Rankin et al., 2005; Fricke et al., 2009). Interestingly, ATM was rarely reported to be resistant in *S.* Newport, and in *Salmonella* isolated from United States. Future studies are needed to investigate if ATM resistance are due to new genes i.e., *bla_PER_* or new gene alleles (Liebana et al., 2004; Srivastava et al., 2014).

### Limitations and Future Prospects

Although using the exact MICs can detect high-resolution, previously under-appreciated bacterial populations, the current study could have some limitations. Firstly, there are patterns of sampling-bias, with more bovine isolates in animal-samplings, and no environmental or fresh produce samples. As mentioned in earlier studies, isolates from diverse animal origins, particularly poultry samples, and other environmental samples is crucial to address *S.* Newport lineage-reservoir issues (Yue and Schifferli, 2014; Yue et al., 2015; De Masi et al., 2017). Secondly, information on international travel for human cases was not available, which is currently considered as a high-risk factor for non-typhoidal *Salmonella* infections. Thirdly variables such as those on geospatial factors, animal husbandry, sampling strategy, sampling framework and population selection have not been considered. Nevertheless, by applying machine-learning algorithm with standardized statistical procedures, this study tries to develop an integrated overview of significant factors (subgroups of antibiotic-resistant phenotype) affecting both animals and humans, so interventions at both clinical- and policy-level can be adjusted simultaneously.

## Author Contributions

HP and NP contributed equally to this work, conducted the data analysis, and drafted the manuscript. XL and WF made significant contribution to refine and reorganized the data used in the manuscript and its presentation. MY conceived the idea, collected the data, and suggested the statistical interpretation of the data analyzed. All authors have read and approved the manuscript.

## Conflict of Interest Statement

The authors declare that the research was conducted in the absence of any commercial or financial relationships that could be construed as a potential conflict of interest.
