# Supplementary material for: Multiple Food-Animal-Borne Route in Transmission of Antibiotic-Resistant Salmonella Newport to Humans
Source: Front Microbiol. 2018 Jan 23;9:23. doi: 10.3389/fmicb.2018.00023 (PMC5787089; doi:10.3389/fmicb.2018.00023)
Supplement: Supplementary file 1 [file Table_1.DOCX]

Supplementary Material

**Multiple food-animal-borne route in transmission of antibiotic-resistant *Salmonella* Newport to humans**

Hang Pan^1$^, Narayan Paudyal^1$^, Xiaoliang Li^1^, Weihuan Fang^1,2^ and Min Yue^1,2*^

*** Correspondence:** Min Yue, [myue@zju.edu.cn](mailto:myue@zju.edu.cn)

***^$^****These authors contributed equally to this work.*

# Supplementary Figures and Tables

**1.2 Supplemental Table 1.** The number of strain in the corresponding each of 149 distinct antibiogram among different hosts.

| Antibiogram Combination | Bovine | Chicken | Porcine | Turkey | Human |
| --- | --- | --- | --- | --- | --- |
| AmcAmpAxoCepChlFoxSmxStrTioTet | 187 | 8 | 5 | 2 | 76 |
| StrTet | 168 | 40 | 16 | 86 | 2214 |
| Tet | 16 | 4 | 5 | 6 | 171 |
| AmcAmpAxoChlFisFoxStrTioTet | 88 | 1 | 1 | 0 | 28 |
| AmcAmpAxoCepChlFoxKanSmxStrTioTet | 24 | 0 | 4 | 0 | 24 |
| AmcAmpAxoCepChlCotFoxSmxStrTioTet | 27 | 0 | 3 | 1 | 7 |
| AmcAmpAxoChlFisFoxKanStrTioTet | 17 | 1 | 0 | 0 | 4 |
| AmcAmpAtmAxoCazChlCtxFisFoxStrTioTet | 14 | 0 | 1 | 0 | 1 |
| ChlSmxStrTet | 10 | 0 | 1 | 0 | 6 |
| AmcAmpAxoCepChlFoxGenSmxStrTioTet | 9 | 0 | 0 | 1 | 3 |
| AmcAmpAxoCazChlFisFoxStrTioTet | 3 | 0 | 1 | 0 | 1 |
| StrTioTet | 1 | 0 | 1 | 1 | 10 |
| AmcAmpAxoCepChlFoxGenKanSmxStrTioTet | 9 | 0 | 0 | 0 | 9 |
| AmcAmpAxoChlCotFisFoxKanStrTioTet | 4 | 0 | 0 | 0 | 1 |
| AmcAmpAxoCepChlFoxNalSmxStrTioTet | 1 | 0 | 0 | 0 | 1 |
| AmcAmpAxoCepChlSmxStrTioTet | 17 | 0 | 0 | 0 | 10 |
| AmcAmpAxoCepChlCotSmxStrTioTet | 1 | 0 | 0 | 0 | 2 |
| AmcAmpAxoChlCipFisFoxNalStrTioTet | 2 | 0 | 0 | 0 | 1 |
| SmxStrTet | 1 | 0 | 0 | 0 | 7 |
| ChlFisStrTet | 27 | 0 | 0 | 0 | 7 |
| AmcAmpChlKanSmxStrTet | 1 | 0 | 0 | 0 | 1 |
| AmpChlSmxStrTet | 1 | 0 | 0 | 0 | 2 |
| AmpFisStrTet | 5 | 0 | 0 | 0 | 3 |
| AmcAmpFisStrTet | 3 | 0 | 0 | 0 | 1 |
| AmpStrTet | 0 | 1 | 0 | 1 | 10 |
| GenSmxStrTet | 0 | 0 | 0 | 8 | 4 |
| FisGenStrTet | 0 | 0 | 0 | 6 | 5 |
| ChlStrTet | 0 | 0 | 1 | 0 | 1 |
| NalStrTet | 0 | 0 | 0 | 1 | 3 |
| GenStrTet | 0 | 1 | 1 | 1 | 5 |
| AmcAmpFisGenKanStrTet | 0 | 0 | 0 | 1 | 1 |
| AmcAmpAxoCepChlFoxGenKanStrTioTet | 1 | 0 | 0 | 1 | 0 |
| AmcAmpAxoChlCotFisFoxGenKanStrTioTet | 1 | 1 | 0 | 1 | 0 |
| AmcAmpAxoCepChlFoxStrTioTet | 13 | 1 | 0 | 0 | 0 |
| AmcAmpAxoChlCotFisFoxStrTioTet | 13 | 1 | 0 | 0 | 0 |
| ChlFisKanStrTet | 1 | 0 | 0 | 1 | 0 |
| AmcAmpChlFisStrTet | 1 | 0 | 0 | 1 | 0 |
| AmcAmpAtmAxoCazCeqChlCipCotCtcCtxFisFoxGenKanPtzStrTioTet | 0 | 0 | 0 | 0 | 1 |
| AmcAmpAtmAxoCazCeqChlCtcCtxFisFoxGenKanPtzStrTioTet | 0 | 0 | 0 | 0 | 1 |
| AmcAmpAxoCepChlCotFoxGenSmxStrTioTet | 0 | 0 | 0 | 0 | 1 |
| AmcAmpAtmAxoCazCeqChlCotCtcCtxFisFoxGenKanPtzStrTioTet | 0 | 0 | 0 | 0 | 2 |
| AmcAmpAtmAxoCazCeqChlCotCtxFisFoxKanPtzStrTioTet | 0 | 0 | 0 | 0 | 1 |
| AmcAmpAtmAxoCazCeqChlCotCtcCtxFisFoxKanPtzStrTioTet | 0 | 0 | 0 | 0 | 1 |
| AmcAmpAtmAxoCazCeqChlCtcCtxFisFoxKanPtzStrTioTet | 0 | 0 | 0 | 0 | 2 |
| AmcAmpAtmAxoCazCeqChlCtcCtxFisFoxKanStrTioTet | 0 | 0 | 0 | 0 | 3 |
| AmcAmpAtmAxoCazCeqChlCtxFisFoxKanPtzStrTioTet | 0 | 0 | 0 | 0 | 2 |
| AmcAmpAtmAxoCazCeqChlCtxFisFoxStrTioTet | 0 | 0 | 0 | 0 | 6 |
| AmcAmpAtmAxoCazCeqChlCtxFisFoxKanStrTioTet | 0 | 0 | 0 | 0 | 1 |
| AmcAmpAxoCazCeqChlFisFoxKanStrTioTet | 0 | 0 | 0 | 0 | 1 |
| AmcAmpAxoCazCeqChlCtxFisFoxStrTioTet | 0 | 0 | 0 | 0 | 2 |
| AmcAmpAxoCepFoxKanSmxStrTioTet | 0 | 0 | 0 | 0 | 1 |
| AmcAmpAxoCazCeqChlFisFoxStrTioTet | 0 | 0 | 0 | 0 | 4 |
| AmcAmpAtmAxoCazCeqChlCtxFisFoxPtzStrTioTet | 0 | 0 | 0 | 0 | 4 |
| AmcAmpAxoCazCeqChlCtcCtxFisFoxStrTioTet | 0 | 0 | 0 | 0 | 1 |
| AmcAmpAtmAxoCazCeqChlCtcCtxFisFoxPtzStrTioTet | 0 | 0 | 0 | 0 | 30 |
| AmcAmpAtmAxoCazCeqChlCtcCtxFisFoxStrTioTet | 0 | 0 | 0 | 0 | 20 |
| AmcAmpAtmAxoCazCeqChlFisFoxStrTioTet | 0 | 0 | 0 | 0 | 2 |
| AmcAmpAtmAxoCazCeqChlCtcFisFoxStrTioTet | 0 | 0 | 0 | 0 | 1 |
| AmcAmpAxoCazCeqChlFisFoxPtzStrTioTet | 0 | 0 | 0 | 0 | 1 |
| AmcAmpAtmAxoCazCeqChlCtxFoxStrTioTet | 0 | 0 | 0 | 0 | 1 |
| AmcAmpAtmAxoCazCeqChlCotCtxFisFoxPtzStrTioTet | 0 | 0 | 0 | 0 | 1 |
| AmcAmpAtmAxoCazCeqChlCotCtcCtxFisFoxPtzStrTioTet | 0 | 0 | 0 | 0 | 4 |
| AmcAmpAtmAxoCazCeqCtcCtxFisFoxPtzStrTioTet | 0 | 0 | 0 | 0 | 1 |
| AmcAmpAxoCepChlCipFoxNalSmxStrTioTet | 0 | 0 | 0 | 0 | 1 |
| AmcAmpAtmAxoCazCeqChlCipCtcCtxFisFoxNalPtzStrTioTet | 0 | 0 | 0 | 0 | 2 |
| AmcAmpAtmAxoCazCeqCipCtcCtxFoxNalStrTio | 0 | 0 | 0 | 0 | 1 |
| AmcAmpAtmAxoCazCeqCipCtcCtxFoxNalStrTioTet | 0 | 0 | 0 | 0 | 2 |
| AmcAmpAxoCazCeqCtcFisFoxPtzStrTioTet | 0 | 0 | 0 | 0 | 1 |
| AmcAmpAtmAxoCazCeqCtcCtxFoxStrTioTet | 0 | 0 | 0 | 0 | 1 |
| AmcAmpAxoImiSmxStrTioTet | 0 | 0 | 0 | 0 | 1 |
| CipNalStrTet | 0 | 0 | 0 | 0 | 7 |
| CipNalTet | 0 | 0 | 0 | 0 | 1 |
| CeqStrTioTet | 0 | 0 | 0 | 0 | 1 |
| FoxTet | 0 | 0 | 0 | 0 | 1 |
| CipStrTet | 0 | 0 | 0 | 0 | 1 |
| CotStrTet | 0 | 0 | 0 | 0 | 1 |
| CotSmxStrTet | 0 | 0 | 0 | 0 | 1 |
| CepChlFoxStrTioTet | 0 | 0 | 0 | 0 | 1 |
| ChlStrTioTet | 0 | 0 | 0 | 0 | 2 |
| ChlCipStrTioTet | 0 | 0 | 0 | 0 | 1 |
| FoxStrTioTet | 0 | 0 | 0 | 0 | 1 |
| ImiStrTioTet | 0 | 0 | 0 | 0 | 1 |
| AmpNalStrTioTet | 0 | 0 | 0 | 0 | 1 |
| FisStrTioTet | 0 | 0 | 0 | 0 | 1 |
| ChlKanSmxStrTet | 0 | 0 | 0 | 0 | 1 |
| ChlCipFisKanStrTioTet | 0 | 0 | 0 | 0 | 1 |
| AmpGenStrTet | 0 | 0 | 0 | 0 | 1 |
| AmcAmpSmxStrTet | 0 | 0 | 0 | 0 | 1 |
| AmcAmpCepSmxStrTet | 0 | 0 | 0 | 0 | 1 |
| AmpChlFisStrTet | 0 | 0 | 0 | 0 | 2 |
| AmpCotFisStrTet | 0 | 0 | 0 | 0 | 1 |
| AmcAmpFoxStrTet | 0 | 0 | 0 | 0 | 1 |
| AmcAmpStrTet | 0 | 0 | 0 | 0 | 3 |
| AmcAmpTet | 0 | 0 | 0 | 0 | 1 |
| AmcAmpCepChlCipGenNalSmxStrTet | 0 | 0 | 0 | 0 | 1 |
| AmpCipCotFisGenKanNalStrTet | 0 | 0 | 0 | 0 | 1 |
| AmcAmpCepChlCotGenSmxStrTet | 0 | 0 | 0 | 0 | 2 |
| AmcAmpCepChlCotGenKanSmxStrTet | 0 | 0 | 0 | 0 | 2 |
| AmpCepChlFoxGenKanSmxStrTioTet | 0 | 0 | 0 | 0 | 1 |
| AmcAmpAxoChlFoxGenStrTioTet | 1 | 0 | 0 | 0 | 0 |
| AmcAmpAxoCepChlFoxGenStrTioTet | 2 | 0 | 0 | 0 | 0 |
| AmcAmpAxoCepChlGenSmxStrTioTet | 1 | 0 | 0 | 0 | 0 |
| AmcAmpAxoCepCotFoxGenKanStrTioTet | 1 | 0 | 0 | 0 | 0 |
| AmcAmpAxoCepChlCotFoxKanSmxStrTioTet | 2 | 0 | 0 | 0 | 0 |
| AmcAmpAxoCepChlCotFoxGenKanSmxStrTioTet | 2 | 0 | 0 | 0 | 0 |
| AmcAmpAxoCepChlCotFoxGenKanStrTioTet | 1 | 0 | 0 | 0 | 0 |
| AmcAmpAxoChlCotFisFoxGenStrTioTet | 1 | 0 | 0 | 0 | 0 |
| AmcAmpAxoChlCotFoxKanSmxStrTioTet | 1 | 0 | 0 | 0 | 0 |
| AmcAmpAxoChlFoxKanSmxStrTioTet | 2 | 0 | 0 | 0 | 0 |
| AmcAmpAtmAxoCazChlCtxFisFoxKanStrTioTet | 2 | 0 | 0 | 0 | 0 |
| AmcAmpAxoCepChlFoxKanStrTioTet | 2 | 0 | 0 | 0 | 0 |
| AmcAmpAxoCepChlCipSmxStrTioTet | 1 | 0 | 0 | 0 | 0 |
| AmcAmpAxoChlFoxSmxStrTioTet | 15 | 0 | 0 | 0 | 0 |
| AmcAmpAtmAxoCazChlCtxFisFoxPtzStrTioTet | 8 | 0 | 0 | 0 | 0 |
| AmcAmpAxoCazChlCtxFisFoxStrTioTet | 2 | 0 | 0 | 0 | 0 |
| AmcAmpAxoCepChlStrTioTet | 1 | 0 | 0 | 0 | 0 |
| AmcAmpAxoChlFoxStrTioTet | 1 | 0 | 0 | 0 | 0 |
| AmcAmpAxoCotFisFoxStrTioTet | 1 | 0 | 0 | 0 | 0 |
| AmcAmpAtmAxoCazChlCotCtxFisFoxStrTioTet | 3 | 0 | 0 | 0 | 0 |
| AmcAmpAxoChlCotFoxSmxStrTioTet | 4 | 0 | 0 | 0 | 0 |
| AmcAmpAxoCepStrTioTet | 1 | 0 | 0 | 0 | 0 |
| AmcAmpAxoCazFisFoxStrTioTet | 1 | 0 | 0 | 0 | 0 |
| AmcAmpAtmAxoCazCtxFisFoxStrTioTet | 1 | 0 | 0 | 0 | 0 |
| AmcAmpAxoFisFoxStrTioTet | 6 | 0 | 0 | 0 | 0 |
| AmcAmpAtmAxoCazFisFoxStrTioTet | 1 | 0 | 0 | 0 | 0 |
| AmcAmpAxoCepFoxSmxStrTioTet | 4 | 0 | 0 | 0 | 0 |
| AmcAmpAxoFoxSmxStrTioTet | 2 | 0 | 0 | 0 | 0 |
| AmcAxoCepChlFoxStrTioTet | 1 | 0 | 0 | 0 | 0 |
| AmcAmpAxoCepChlCipFoxNalStrTioTet | 1 | 0 | 0 | 0 | 0 |
| AmcAmpAtmAxoCazChlCipCtxFisFoxNalStrTioTet | 1 | 0 | 0 | 0 | 0 |
| AmcAmpAxoCepFoxStrTioTet | 1 | 0 | 0 | 0 | 0 |
| CepStrTet | 1 | 0 | 0 | 0 | 0 |
| ChlCotFisGenStrTet | 1 | 0 | 0 | 0 | 0 |
| ChlCotSmxStrTet | 1 | 0 | 0 | 0 | 0 |
| AmpSmxStrTet | 2 | 0 | 0 | 0 | 0 |
| AmcAmpAtmAxoCazCtxFisFoxGenKanStrTioTet | 0 | 0 | 0 | 2 | 0 |
| AmcAmpAxoFoxGenKanStrTioTet | 0 | 0 | 0 | 1 | 0 |
| AmcAmpAxoFisFoxGenKanStrTioTet | 0 | 0 | 0 | 2 | 0 |
| AmcAmpAtmAxoCazCtxFisFoxGenKanPtzStrTioTet | 0 | 0 | 0 | 1 | 0 |
| AmcAmpAxoFoxStrTioTet | 0 | 0 | 0 | 1 | 0 |
| AmcAmpAtmAxoCazCtxFoxKanStrTioTet | 0 | 0 | 0 | 1 | 0 |
| KanStrTet | 0 | 0 | 0 | 2 | 0 |
| FisGenKanStrTet | 0 | 0 | 0 | 3 | 0 |
| GenKanSmxStrTet | 0 | 0 | 0 | 1 | 0 |
| ChlGenKanSmxStrTet | 0 | 0 | 0 | 2 | 0 |
| AmcAmpCepGenSmxStrTet | 0 | 1 | 0 | 0 | 0 |
| AmpFisGenStrTet | 0 | 0 | 0 | 1 | 0 |
| AmpFisGenKanStrTet | 0 | 0 | 0 | 1 | 0 |
| AmpCotStrTet | 0 | 0 | 0 | 1 | 0 |
